# Functional Characterization of a Drought-Responsive Invertase Inhibitor from Maize (*Zea mays* L.)

**DOI:** 10.3390/ijms20174081

**Published:** 2019-08-21

**Authors:** Lin Chen, Xiaohong Liu, Xiaojia Huang, Wei Luo, Yuming Long, Steffen Greiner, Thomas Rausch, Hongbo Zhao

**Affiliations:** 1 College of Horticulture, South China Agricultural University, Guangzhou 510642, China; 2 Centre for Organismal Studies Heidelberg, Department of Plant Molecular Physiology, University of Heidelberg, 69120 Heidelberg, Germany

**Keywords:** *Zea mays*, invertase inhibitor, drought, abscisic acid (ABA), regulation

## Abstract

Invertases (INVs) play essential roles in plant growth in response to environmental cues. Previous work showed that plant invertases can be post-translationally regulated by small protein inhibitors (INVINHs). Here, this study characterizes a proteinaceous inhibitor of INVs in maize (Zm-INVINH4). A functional analysis of the recombinant Zm-INVINH4 protein revealed that it inhibited both cell wall and vacuolar invertase activities from maize leaves. A Zm-INVINH4::green fluorescent protein fusion experiment indicated that this protein localized in the apoplast. Transcript analysis showed that *Zm-INVINH4* is specifically expressed in maize sink tissues, such as the base part of the leaves and young kernels. Moreover, drought stress perturbation significantly induced *Zm-INVINH4* expression, which was accompanied with a decrease of cell wall invertase (CWI) activities and an increase of sucrose accumulation in both base parts of the leaves 2 to 7 days after pollinated kernels. In summary, the results support the hypothesis that INV-related sink growth in response to drought treatment is (partially) caused by a silencing of INV activity via drought-induced induction of Zm-INVINH4 protein.

## 1. Introduction

*Zea mays L. ssp. Mays* is one of the world’s most important cereal plants. Sucrose as the transport form of carbohydrates in maize is synthesized in source-leaf mesophyll cells and transported through the phloem to develop sink organs by “mass flow” [1,2]. Adjustment of source-to-sink resource allocation can impact on grain filling, and thus it is very important in maize yield improvement [3]. The source-to-sink adjustment in plants is not only affected by endogenous factors, such as the competition between different sink organs, but also exogenous factors, for example, abiotic stress or pathogen infection [4,5]. An organ’s “sink strength” is dependent upon a number of factors, including the localized osmotic status and the activity of the sucrose-cleaving enzymes [6,7].

Acid invertase, a sucrose-cleaving enzyme which produces hexose sugars from sucrose, regulates the plant primary carbon metabolism and effects on plant growth and development [8,9]. In maize, acid invertases regulate cell divisions of the endosperm and embryo, especially during the early kernel development [10]. Based on subcellular location difference, acid invertases can be grouped into vacuolar invertase and cell wall invertase. Some acid invertase in maize, such as cell wall invertase (Zm-INCW2) and a vacuolar invertase (Zm-IVR2) have been found playing significant roles in sucrose partitioning and sink development and provide hexoses to support maize cell division [11,12].

Acid invertases display multiple regulatory mechanisms at the transcriptional and post-transcriptional levels in response to environmental stimuli, such as drought [13], temperature stress [14], salinity [15], and abscisic acid (ABA) [16], etc. The presence of endogenous small (<20 kDa) inhibitor proteins (INVINH) could post-translationally inhibit invertase activity at certain stages of plant development [17].

Drought effecting source-sink regulation is a major factor limiting plant distribution and productivity. In maize, the pre- and early post-pollination phases of female reproductive development are critical for sufficient pollination and seed set. The process is extremely sensitive to drought [18,19]. Drought could negatively affect photosynthesis and thus alter the whole plant carbohydrate status, finally resulting in losses in grain yield or seed abortion [20,21]. However, exogenous sugar injection in the maize stem has been found to be able to induce the invertase inhibitor activities and decrease the maize abortion ratio under drought conditions [18]. It is suggested that invertase inhibitors could ameliorate the effects of drought by regulating the sugar metabolism.

Previously, an invertase inhibitor, named Zm-INVINH1, localized to the embryo surrounding the region during early kernel development, has been found, and its enzyme activity was identified in vitro [22]. However, several questions have remained: Does maize have additional INVINHs isoforms? Are INVINHs drought responsive? Is INV-related maize sink growth in response to drought stress caused by the silencing of INV activity via induction of INVINH proteins?

Here, this research presents an apoplast-localized proteinaceous inhibitor of INV in maize, named Zm-INVINH4, which has inhibitory activity by reducing maize invertase activity and is specifically expressed in the base part of leaves and young kernels after drought stimulus, accompanied by the decrease of cell wall invertase (CWI) activities and the increase of sucrose accumulation. Results also suggest that Zm-INVINH4 may modulate the INV-related source to sink growth and the development in maize under the drought stress regime.

## 2. Results

### 2.1. Identification of the Maize Invertase Inhibitors

**Figure 1:**
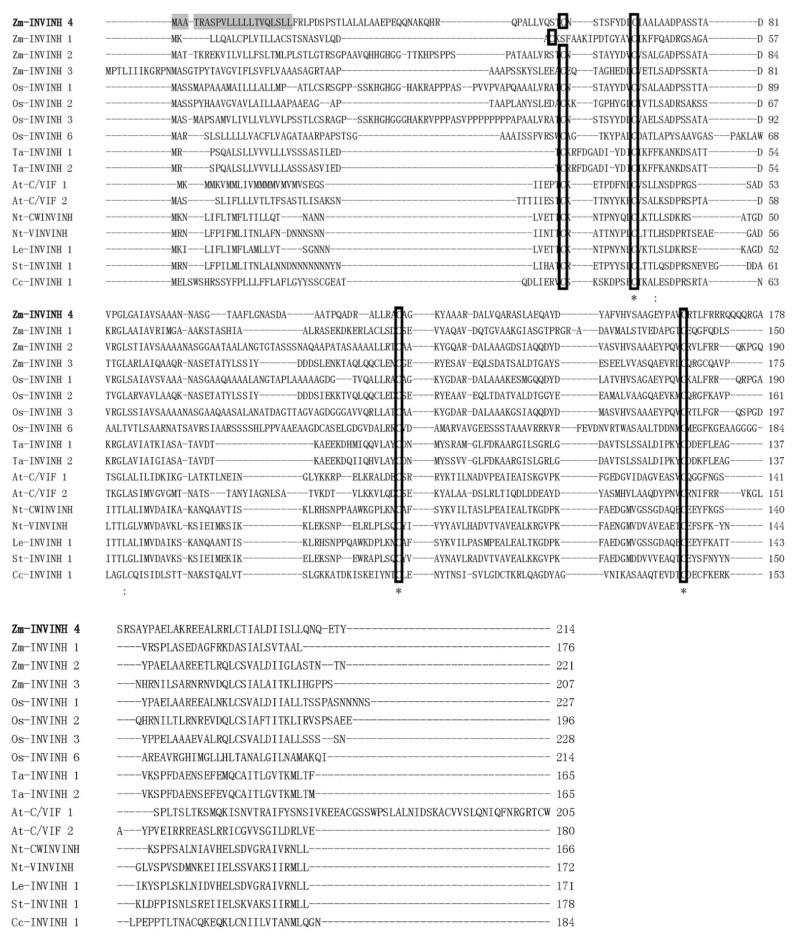
Comparison of the maize cell wall invertase inhibitor 4 (Zm-INVINH4) protein sequence with other invertase inhibitor-like proteins. Alignment of the deduced amino acid sequence of INVINH-like proteins from maize, rice, wheat, Arabidopsis, tobacco, tomato, potato, and coffee. The conserved four Cys residues are indicated by the rectangle. Identical and conserved residues are marked with asterisks and dots/colons, respectively. Shaded regions indicate the potential signal peptide cleavage site of Zm-INVINH4. The accession numbers of the sequences used to construct the alignment were: Y12805 (tobacco cell wall invertase inhibitor (Nt-CWINVINH)), Y12806 (tobacco vacuolar invertase inhibitor (Nt-VINVINH)), NM_103692 (Arabidopsis cell wall/vacuolar invertase inhibitor 1 (At-C/VIF1)), NM_125858 (At-C/VIF2, Arabidopsis cell wall/vacuolar invertase inhibitor 2), GRMZM2G162447 (maize cell wall invertase inhibitor 1 (Zm-INVINH1)), AX214336 (maize cell wall invertase inhibitor 2 (Zm-INVINH2)), AX214357 (maize cell wall invertase inhibitor 3 (Zm-INVINH3)), AJ010943 (tomato invertase inhibitor (Le-INVINH1)), NM_001067338 (rice invertase inhibitor 1 (Os-INVINH1)), NM_001049399 (rice invertase inhibitor 2 (Os-INVINH2)), AX214360 (rice invertase inhibitor 3 (Os-INVINH3)), NM_001071560 (rice invertase inhibitor 6 (Os-INVINH6)), FJ810208 (potato invertase inhibitor (St-INVINH1)), DQ834320 (coffee invertase inhibitor (Cc-INVINH1)). Two wheat invertase inhibitor (Ta-INVINH1 and Ta-INVINH2) amino acid sequences were found in the paper [22].

To search invertase inhibitor homologs and elucidate the role of cell walls or/and vacuolar invertase inhibitors in maize, previously characterized monocot invertase inhibitor sequences were used as queries to search in the maize genome systematically with a basic local alignment search tool (BLAST). One putative maize invertase inhibitor sequence was identified. The sequence has a similar size and shared sequence homology with monocot INVINH proteins and was named *Zm-INVINH4* (GenBank accession no: NM_001159249). It is located in chromosome 2 with 642 nucleotides in the open reading frame (Figure 1).

A deduced amino acid sequence of Zm-INVINH4 was aligned with previously reported genes from other species using the CLUSTAL W2 program [23]. The result of the comparison of Zm-INVINH4 with INVINH from other species showed that there was four conserved Cys residues in all of the sequences (Figure 1), which was a hallmark of all known plant invertase inhibitors [17].

Phylogenetic and molecular evolutionary analysis was conducted with MEGA 5.05 [24]. Amino acid sequences of pectin methylesterase inhibitors (PMEIs) were added as: Arabidopsis PMEI 1 (AT1G48020), Arabidopsis PMEI 2 (AT1G48020), and *Actinidia deliciosa* PMEI (AAZ20131). A comparison of these with the closest homologs in the *Poaceae* species reveals a higher degree of similarity of PMEIs between maize and *Poaceae* than other species.

**Figure 2:**
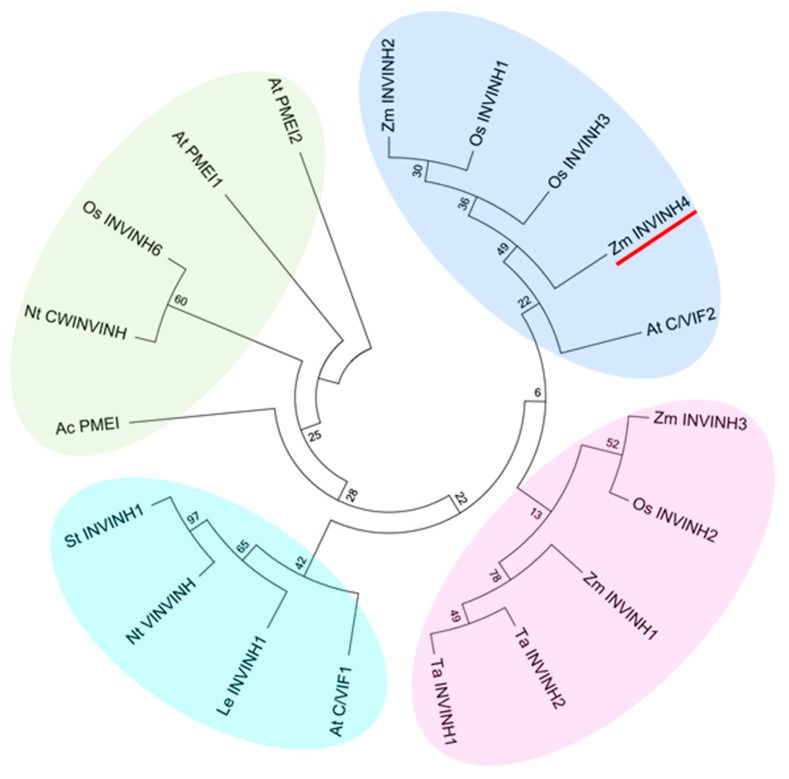
Phylogenetic analysis of invertase inhibitors of plants based on the predicted amino acid sequences. Four groups can be discerned. Bootstrap values (%) are indicated for major branches. Zm-INVINH4 was marked with red line. Abbreviations for the species are: *Actinidia deliciosa* (Ac); *Arabidopsis thaliana* (At); *Coffea canephora* (Cc); *Nicotiana tabacum* (Nt); *Lycopersicum esculentum* (Le); *Oryza sativa* (Os); *Solanum tuberosum* (St); *Triticum aestivum* (Ta); *Zea mays* (Zm).

In addition, Zm-INVINH4 clustered most closely with rice apoplastic invertase inhibitors and the Arabidopsis cell wall and vacuolar invertase inhibitor 2 (Figure 2). Furthermore, Zm-INVINH4 was distant with those from dicot, such as tobacco, potato, and tomato (Figure 2).

Full-length cDNA of the *Zm-INVINH4* was cloned from maize leaves and young kernels. The protein was 20.36 KD with a pI value of 7.81, which is in accordance with other INVINH-related proteins. There was an apparent signal peptide sequence with 21 amino acid residues in Zm-INVINH4 protein.

### 2.2. Characterization of the Zm-INVINH4 Recombinant Protein

**Figure 3:**
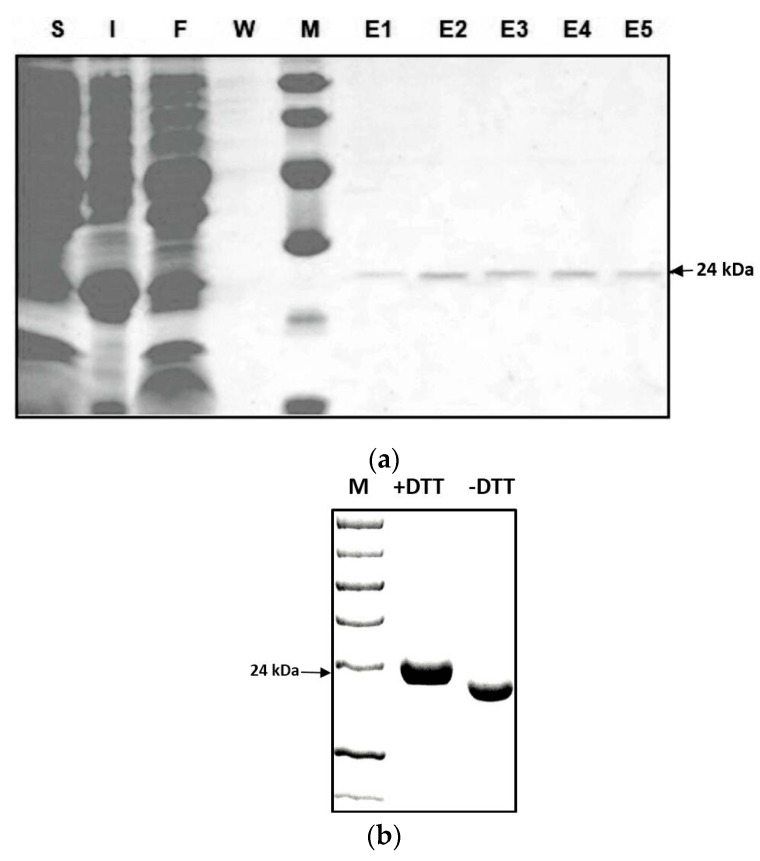
(**a**) SDS–PAGE of the recombinant Zm-INVINH4 protein in *E.coli* with a predicted mass of 24 kDa. Soluble proteins (S); insoluble proteins (I); flow-through of the column (F); wash fraction (W); and elution (E1–5). (**b**) The increased mobility of the recombinant Zm-INVINH4 protein during gel electrophoresis in the absence of a reductant, as compared with the reductant (DTT)-containing sample buffer, which indicates that the active conformation forms a rather compact structure.

To identify whether Zm-INVINH4 functions as an invertase inhibitor in vitro, a partial cDNA with the predicted mature protein region of Zm-INVINH4, i.e., lacking the signal peptide (Figure 1), was ligated in-frame and was heterologously expressed in *E. coli* expression system. The recombinant Zm-INVINH4 with 6xHis-tag showed the expected molecular mass of 24 kDa on the SDS-PAGE gel (Figure 3a). According to previous knowledge [25], considering that the 6xHis-tag does not affect the recombinant invertase inhibitor protein’s activity, the tag was not cleaved by the Tobacco Etch Virus (TEV) protease in the following experiment. The Zm-INVINH4 possesses four cysteine residues, which are conserved and are important for the formation of two disulfide bridges, making the protein sensitive to treatment with reducing agents, such as DL-Dithiothreito (DTT). Analysis of Zm-INVINH4 protein pre-treated with DTT as compared to non-treated protein clearly shows that the correctly folded Zm-INVINH4 recombinant protein has an active and compact structure, due to its two intramolecular disulfide bridges (Figure 3b).

**Figure 4:**
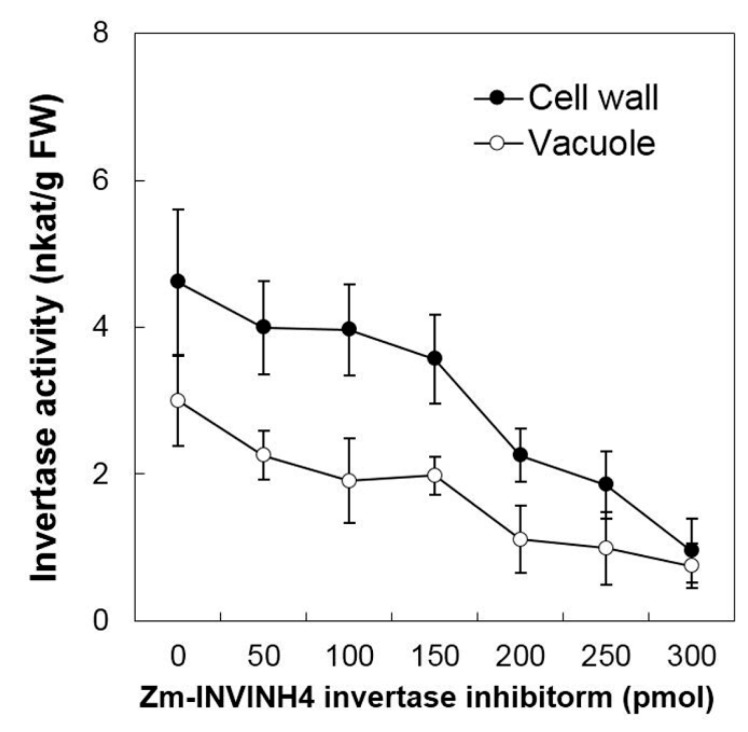
Recombinant Zm-INVINH4 inhibits invertase activity in vitro. Inhibitory activity of the cell wall and vacuolar invertases from maize leaf extracts.

Then the current study analyzed the ability of Zm-INVINH4 recombinant protein to reduce invertase activity in both the cell wall and vacuolar enzyme preparations from the sink part of leaves (Figure 4). Results demonstrated that increasing the Zm-INVINH4 concentration in the invertase reactions resulted in a quantitative decrease in both the cell wall and vacuolar invertase activities. The presence of 300 pmol of inhibitor proteins particularly reduced the leaf cell wall invertase activity by more than three-fold and the vacuolar invertase activity by more than two-fold (Figure 4).

### 2.3. Transient Expression Assay of Zm-INVINH4 in Potato Leaves

**Figure 5:**
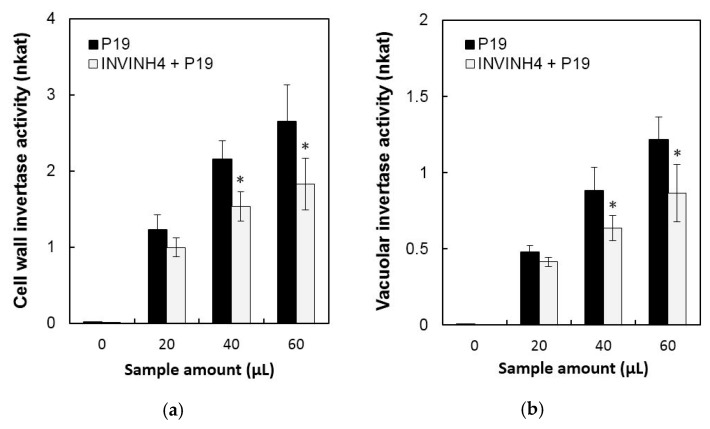
Transient expression assay of Zm-INVINH4 in potato leaves. (**a**) Effect on cell wall invertase activities. (**b**) Effect on vacuolar invertase activities. The enzyme activity data represent mean ± SE of at least four independent biological replicates and asterisks, indicating significant differences in comparison with the P19 by using a Student’s *t*-test: * *p* < 0.05.

In order to further characterize the function of Zm-INVINH4, the method of transient expression by Agrobacteria leaf infiltration in potato leaves was used. *Agrobacterium tumefaciens* cell-suspensions were adjusted to OD 1 with an infiltration buffer and co-infiltrated with a culture harboring the viral suppresser of silencing P19 into the lower epidermis of young potato leaves [25,26]. Leaf proteins were extracted 48 h after infiltration, from which the cell wall and vacuolar invertase activities were detected. The result of the transient expression assay of Zm-INVINH4 in potato leaves showed that both cell wall and vacuolar invertase displayed a significant decline in activity (Figure 5).

### 2.4. Subcellular Localization of Zm-INVINH4

PSORT, Target P, and SIGNAL P were used to predict the subcellular location of Zm-INVINH4. The results suggested that the Zm-INVINH4 protein was targeted to the apoplast (Table 1).

**Figure 6:**
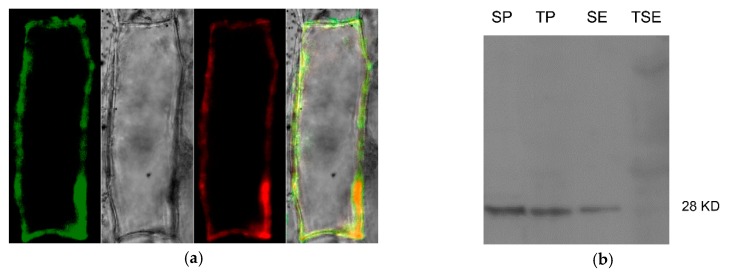
Zm-INVINH4 is targeted in the apoplast. (**a**)Transient expression of Zm-INVINH4::GFP and Zm-INVINH1::RFP under the control of the cauliflower mosaic virus 35S promoter in onion epidermal cells. (**b**) Immunoblot of Zm-INVINH4::HA by using aiti HA-tag antiserum in transient expressed *Nicotiana benthamiana* leaves. SP, soluble fraction; TP, crude pellet immediately boiled; SE, salt elution; TSE, insoluble pellet after salt elution.

A cauliflower mosaic virus 35S promoter controlling Zm-INVINH4::GFP fusion protein construct was generated for transient expression in onion epidermal cells. As Zm-INVINH1 had been proved to be localized in the cell wall [22], the Zm_INVINH1::RFP was taken as a fluorescence reference gene, co-localized with Zm-INVINH4::GFP in a transient expression in the onion epidermal cells, revealing the fluorescence was restricted to the outer edges of the onion cells (Figure 6a). Furthermore, enhanced fluorescence signals have been observed by neutral pH, while fluorescence signals were quenched by low pH, supporting the apoplast targeting of Zm-INVHIN4 [27]. Additional immunoblot work by Zm-INVINH4::HA was accomplished by using anti HA-tag antiserum in transient expressed *Nicotiana benthamiana* leaves. The results showed that a signal appeared in the salt eluted cell wall fractions (Figure 6b).

### 2.5. Expression Pattern of Zm-INVINH4 in Different Maize Tissues under Drought and ABA Stress

**Figure 7:**
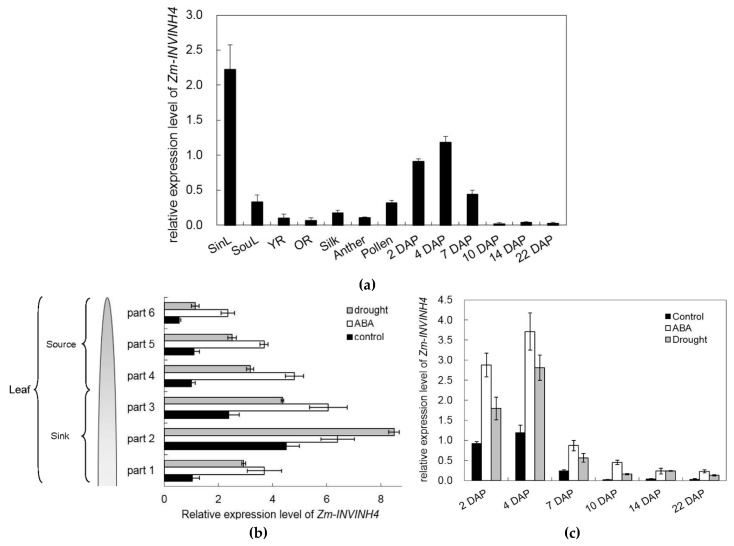
(**a**) Relative expression level of *Zm-INVINH4* in different tissues of *Zea mays*. (**b**) Relative expression level of *Zm-INVINH4* in the source and sink part of the leaf under the drought or ABA treatment. (**c**) Relative expression level of *Zm-INVINH4* in young kernels under the drought or ABA treatment. Sink part of leaf (SinL); source part of leaf (SouL); young root (YR); old root (OR); day after pollination (DAP).

To identify the spatial and temporal expression pattern of *Zm-INVINH4*, RNA was isolated from the leaf, root, stem, silk, anther, and pollen, as well as from a range of early kernel development time points for real-time PCR. The current study results showed that *Zm-INVINH4* was expressed in both vegetative and reproductive tissues (Figure 7a). High expression of the gene was observed in leaves and young kernels, while there was little expression in the root, silk, and anther (Figure 7a). Notably, its mRNA level increased as leaves progressed from source to sink stages. The seed developed from the time of pollination to the 7th day. The expression level decreased sharply (Figure 7a).

To further clarify the spatial and temporal pattern of *Zm-INVINH4*, expression in the source and sink part of the leaf under normal, drought, and ABA stress, each leaf was divided into six parts for analysis. Results showed that drought and ABA significantly induced *Zm-INVINH4* expression in both the source and sink part of the leaf (Figure 7b). In addition, drought stress stimulation of *ZmINVINH4* expression was also observed in young kernels, indicating that *Zm-INVINH4* was up-regulated by drought and ABA dominantly (Figure 7c).

### 2.6. Invertase Activities, Soluble Sugar Accumulation, and Related Gene Expression in the Sink Part of the Leaf

**Figure 8:**
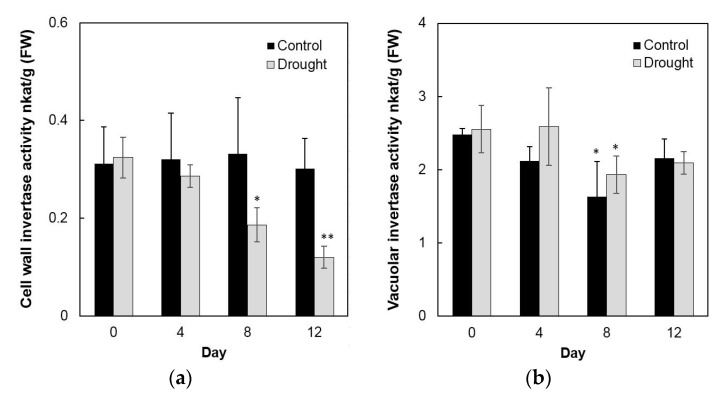
Effect of drought stress on cell wall invertase activities (**a**) and vacuolar invertase activities (**b**) in the maize sink part of the leaf. Data represents mean ± SE of at least four independent biological replicates. Asterisks indicate significant differences in comparison with the control of 0 day by using a Student’s *t*-test: * *p* < 0.05, ** *p* < 0.01.

**Figure 9:**
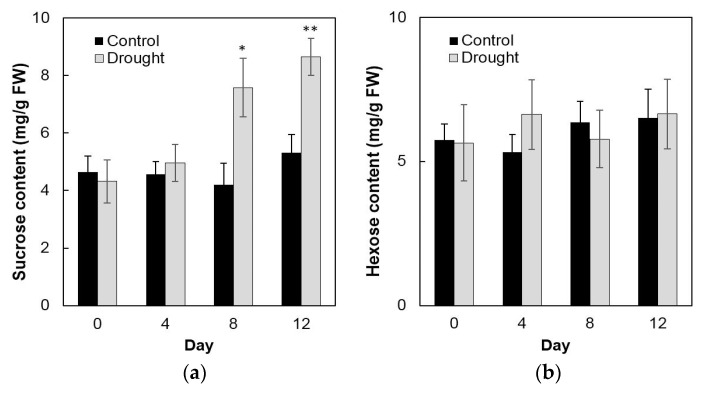
Effect of drought stress on sucrose content (**a**) and hexose content (**b**) in the maize sink part of the leaf. Data of sugar quantification represents mean ± SE of at least six biological replicates. Asterisks indicate significant differences in comparison with the control of 0 day by using a Student’s *t*-test: * *p* < 0.05, ** *p* < 0.01.

To analyze enzyme activities in response to drought stress in the sink part of the leaf, cell wall invertase and vacuolar invertase were extracted drought-treated plants and measured in vitro. The results showed that in the sink part of the leaf vacuolar invertase activities were about 10-fold higher than the cell wall invertase activities (Figure 8a,b). Under drought stress, cell wall invertase activities decreased sharply as compared with the control, as up to 1.9-fold at the 12th day (Figure 8a), whereas vacuolar invertase activities almost remained stable from 0 to 12 days (Figure 8b). This indicated that in the sink part of the leaf only the cell wall invertase responds to a drought-induced stress response. Together with the decrease of cell wall invertase activities, sucrose content analysis showed an accumulation from the 8th day after drought stress and increased about 65% more than the control till the 12th day (Figure 9a). For the hexose content, no significant changes were found during the whole drought treatment (Figure 9b).

**Figure 10:**
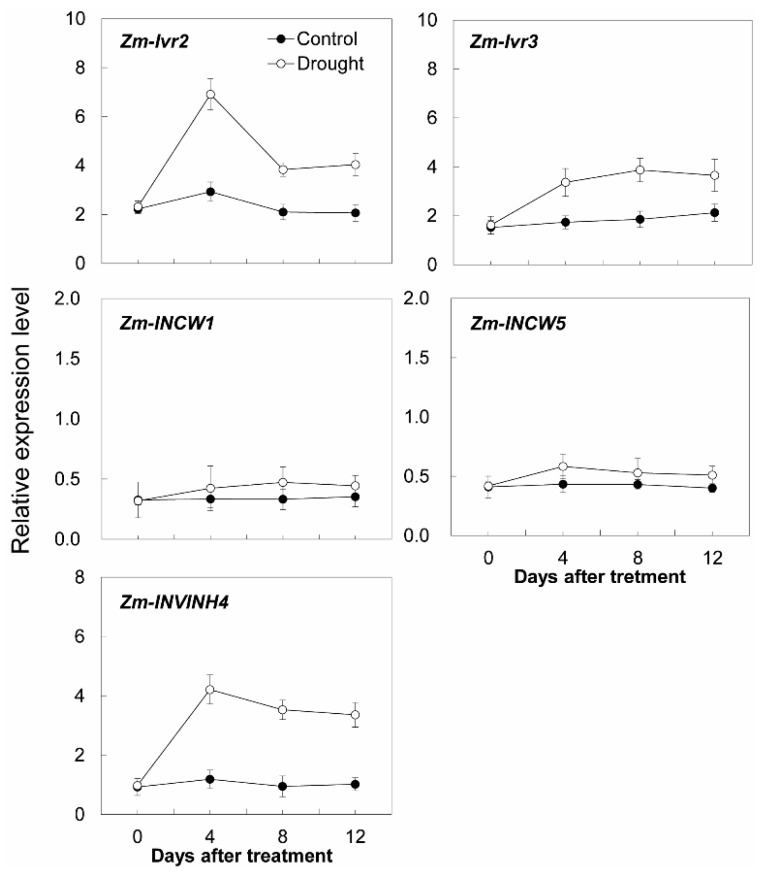
Effect of drought stress on relative expression of invertase and invertase inhibitor-related genes in the maize sink part of the leaf. Gene expression levels were determined by quantitative real-time PCR and were normalized against the maize actin and ubiquitin genes. Data represents mean ± SE of at least three independent biological replicates.

Additional transcript analysis was performed for two cell wall invertase genes (*Zm-INCW1* and *Zm-INCW5*), two vacuolar invertase genes (*Zm-Ivr2* and *Zm-Ivr3*), and *Zm-INVINH4* (Figure 10). Remarkably, Zm-INVINH4, Zm-Ivr2, and Zm-Ivr3 were significantly up-regulated, following the time course of the drought treatment, whereas the expression of *Zm-INCW1* and *Zm-INCW5* was barely affected (Figure 10).

### 2.7. Effects of Invertase Activities, Soluble Sugar Accumulation, and Related Genes Expression in Young Kernels

**Figure 11:**
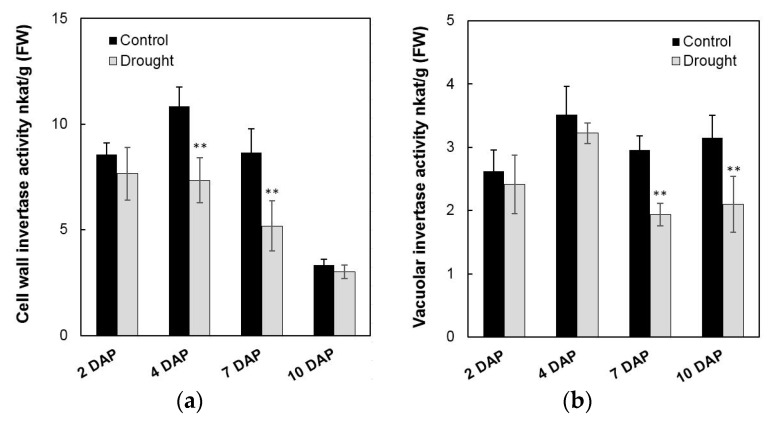
Effect of drought stress on cell wall invertase activities (**a**) and vacuolar invertase activities (**b**) in young maize kernels. Data represents mean ± SE of at least four independent biological replicates. Asterisks indicate significant differences in comparison with the control using a Student’s *t*-test: ** *p* < 0.01.

**Figure 12:**
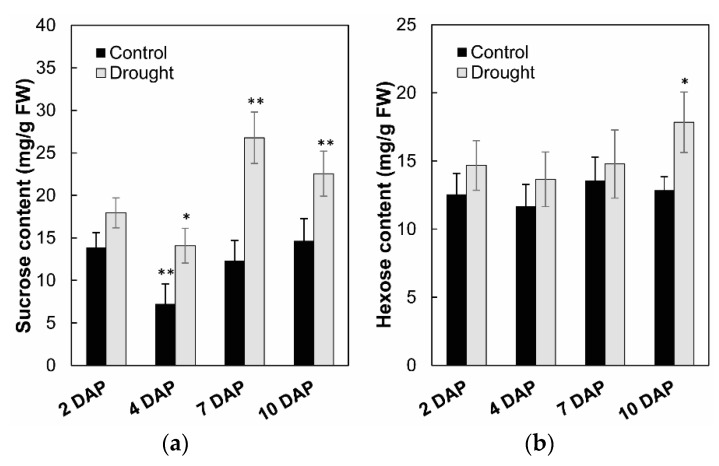
Effect of drought stress on sucrose content (**a**) and hexose content (**b**) in young maize kernels. Data of sugar quantification represents mean ± SE of at least six biological replicates. Asterisks indicate significant differences in comparison with the control using a Student’s *t*-test: * *p* < 0.05, ** *p* < 0.01.

To analyze the effects on enzyme activities in response to drought stress during maize kernel development, cell wall invertase as well as vacuolar invertase were extracted and were measured in vitro. Measurement of enzyme activities showed that cell wall invertase activities were higher than the vacuolar invertase activities in young kernels (Figure 11a,b). Under drought stress, cell wall invertase activities were significantly decreased comparing with the control, especially at 4 to 7 days after pollination of the kernel (Figure 11a). Vacuolar invertase activities decreased significantly from 7 to 10 days after pollination (Figure 11b), indicating that both the cell wall and vacuolar invertases were involved in the drought-induced stress response. Consistent with the decrease of invertase activities, sucrose content analysis showed an accumulation in the kernel from 4 to 10 days after pollination under drought stress, (Figure 12a), whereas for hexose content, no significant changes were observed during the entire drought treatment (Figure 12b).

**Figure 13:**
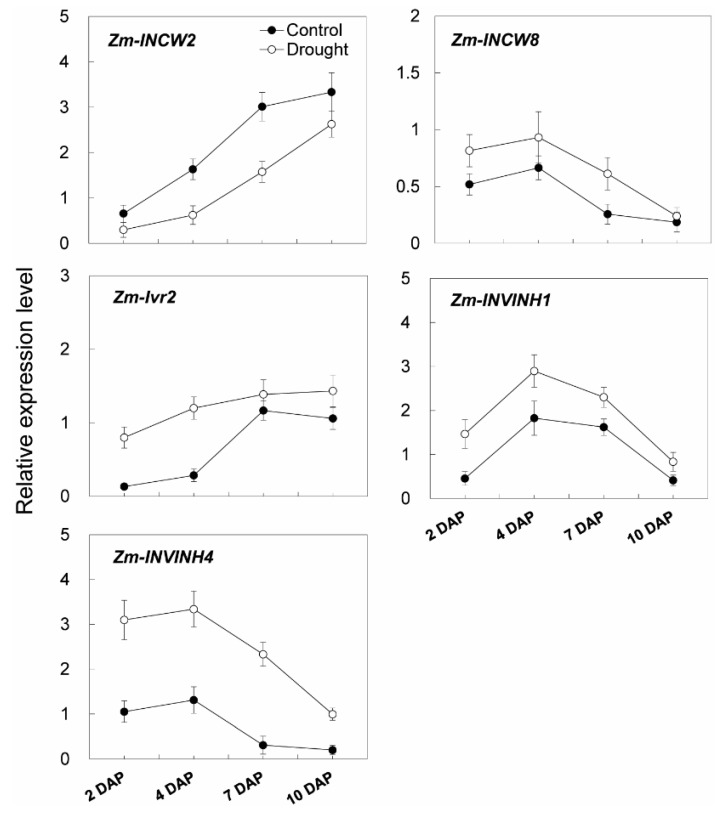
Relative expression of invertase and invertase inhibitor related genes in young maize kernels during drought stress. Gene expression levels were determined by quantitative real-time PCR and were normalized against the maize actin and ubiquitin genes. Data represents mean ± SE of at least three independent biological replicates.

Additional transcript analysis of young kernel-expressed invertase-related genes was carried out. One putative cell wall invertase gene (*Zm-INCW8*), one vacuolar invertase gene (*Zm-Ivr2*), and two inhibitor genes (*Zm-INVINH1* and *Zm-INVINH4*) were found to be constantly induced in young maize kernels during the drought treatment (Figure 13). In marked contrast, a young kernel specifically expressed cell wall invertase gene, *Zm-INCW2*, was down-regulated in response to drought (Figure 13).

## 3. Discussion

In several plant species, it has been shown that invertase inhibitors were encoded by a small gene family [17]. An invertase inhibitor can post-translationally silence invertase activity at certain stages of plant development. A cDNA—encoding putative inhibitor protein in maize, Zm-INVINH1, was expressed especially during 4 to 7 days after pollination in kernels [22]. For maize invertase inhibitor homologs ZmM-INVINH2 and Zm-INVINH3, except the protein sequences that were previously published [22], there was no more information provided. The current study systematically screened the maize genome by using previously characterized monocot invertase inhibitor sequences as the queries. The result indicated that Zm-INVINH4 shares sequence homology with monocot INVINH proteins, and the gene contained four conserved cystine residues, which is a hallmark of all known plant invertase inhibitors. The result of phylogenetic and molecular evolutionary analysis indicated that Zm-INVINH4 was clustered most closely with the rice apoplastic invertase inhibitor and the Arabidopsis cell wall and vacuolar invertase inhibitor 2. In addition, Zm-INVINH4 was distantly with those from dicot, such as tobacco, potato, and tomato.

However, the sequence comparison of invertase inhibitors are closely related to the pectin methylesterase inhibitor (PMEI) proteins [28,29]. Therefore, using a sequence to accurately predict the Zm-INVINH4 function is not recommended. Functional identification by using recombinant protein showed Zm-INVNH4 inhibited both the cell wall and vacuolar invertases in a dose-dependent manner and proved it is an invertase inhibitor. Additional in vivo experiments on potato leaves also showed that Zm-INVINH4 had an invertase inhibitor function. To exclude the possibility of PMEI activities of Zm-INVINH4, a pectin methylesterase activity inhibition experiment was performed by adding recombinant Zm-INVINH4 protein. The result showed that Zm-INVINH4 exhibited none of the PMEI activities (results not shown). Taken together, a biochemical measurement indicated that Zm-INVINH4 is a novel invertase inhibitor in maize.

*Zm-INVINH4* is expressed specifically in maize sink tissues, such as young kernels and base part of leaves under drought stress, indicating that Zm-INVINH4 is a drought-response invertase inhibitor. The question is, which invertase can be inhibited by Zm-INVINH4?

In kernels, from 4 to 10 days after pollination, the current study showed both cell wall and vacuolar invertase activities were decreased in response to drought stress, correlating with sucrose accumulation (Figure 11 and Figure 12).

**Figure 14:**
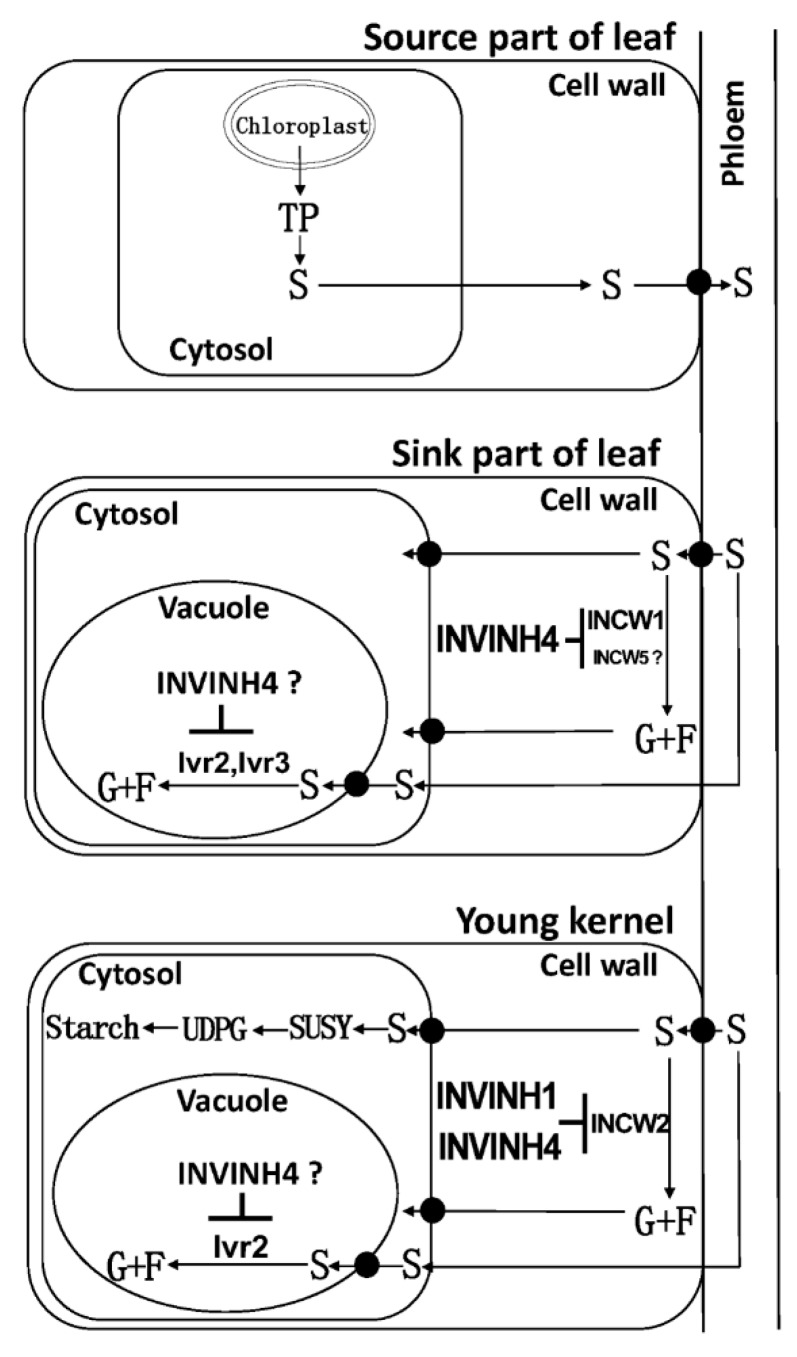
A model explaining the influence of Zm-INVINH4-involved invertase-mediated sucrose degradation from the source to sink in maize. Sucrose (S); fructose (F); glucose (G); cell wall invertase (INCW); vacuolar invertase (Ivr); invertase inhibitor (INVINH); chloroplast-derived triosephosphate (TP); uridine diphosphate glucose (UDPG); sucrose synthase (SUSY). “┴” means inhibition.

The cell wall invertase (CWI) is essential for sucrose partitioning and seed and fruit development. In maize, cell wall invertases have been implicated in early maize floral development, and especially ovary expansion and seed filling [30,31,32]. For cell wall invertase isoforms, the expressions of *Zm-INCW1, Zm-INCW3*, and *Zm-INCW5* were almost undetectable. On the contrary, *Zm-INCW2* was expressed significantly in kernels. Previously, *Zm-INCW2*, a kernel-specific cell wall invertase, has been implicated in early maize floral development, and especially in the ovary expansion [31,32]. A miniature1 mutant with Zm-INCW2 activity loss has shown the seed biomass decreasing by about 70% [30,31,32]. Through a genome-wide survey of invertase encoding genes in maize, the current study also identified a kernel-expressed putative cell wall invertase, named Zm-INCW8. However, it is not an invertase but fructan exohydrolase (data not shown). As Zm-INCW2 is responsible for the cell wall invertase activities during young kernel development, it may be assumed that the apoplast-localized Zm-INVINH4 may bind Zm-INCW2 to form a complex and thereby inhibit the cell wall invertase activities during the young kernel development (Figure 14). Also, *Zm-INVINH1* is specifically expressed in kernels between 4 and 7 days after pollination [25], however, in situ hybridization indicated no expression in basal endosperm transfer cells where Zm-INCW2 expression is localized [25].

In the base part of leaves, another sink tissue with high *Zm-INVINH4* expression, the results suggest that the cell wall invertase Zm-INCW1, a vacuolar invertase Zm-Ivr2 and a putative vacuolar invertase Zm-Ivr3, might bind Zm-INVINH4 to form INV/INVINH complexes, following the time course during the drought treatment. Zm-INVINH4 finally prohibited the cell wall and vacuolar invertase activities (Figure 14). Whether Zm-INCW5, a putative cell wall invertase in leaves, could interact with Zm-INVINH4 is still unclear.

In conclusion, this work provides the evidence that Zm-INVINH4 could function as an inhibitor that prevents invertase activity. Furthermore, it is specifically expressed in the base part of leaves and young kernels during the drought stress, accompanied by a decrease of cell wall invertase (CWI) activities and an increase of sucrose accumulation. This research will help to unravel how Zm-INVINH4 may modulate the invertase (INV)-related source to sink growth and the development in maize under the drought stress regime. Moreover, this research may motivate new strategies on how to improve productivity and avoiding drought-induced seed abortion in maize.

## 4. Materials and Methods

### 4.1. Plant Material and Cultivation

*Zea mays* L. (SEVERUS, KWS, Germany) was grown in the greenhouse on soil at 25 ± 2 °C under long day conditions (16 h light period; 300 μmol m^−2^ s^−1^). Drought treatments were carried out on the three weeks growing seedlings (they were not watered for two weeks) or on the manual pollination stage after flowering for one month. ABA treatment was performed on three weeks seedlings with a final ABA concentration of 100 μM for two weeks. For monitoring gene expression and invertase activity, samples at different times, as indicated in the results, were collected.

Potato (Desiree, from HZAU, China) was grown in the greenhouse at 25 ± 2 °C under long day conditions (16 h light period; 300 μmol m^−2^ s^−1^). The 4th leaves from one month potato plants were used for further experiments.

Plant tissue samples were either used immediately or frozen in liquid nitrogen and stored at −80 °C until used.

### 4.2. Preparation of RNA, cDNA Synthesis, and cDNA Cloning

Total RNA was extracted with the GeneMATRIX Universial RNA purification Kit (Roboklon, Berlin, Germany). cDNA synthesis was performed immediately after DNase (AppliChem, Darmstadt, Germany) treatment, using AMV—Reverse Transcriptase (Roboklon, Berlin, Germany). Full-length cDNAs of Zm-INVINH4 were cloned from maize leaf cDNA by PCR (35 cycles: 95 °C/30 sec–55 °C/30 sec–72 °C/1 min/1 kb; final extension: 10 min), using Phusion High-Fidelity DNA polymerase with a GC buffer (Finnzymes, Schwerte, Germany) and corresponding primers presented in Table S1. PCR products were fully sequenced (Starseq, Heidelberg, Germany) and cloned into a pDONR201 vector (Invitrogen, Darmstadt, Germany) to obtain the entry clone for the Gateway system (Invitrogen, Darmstadt, Germany).

### 4.3. Plasmid Cloning

Recombinant Zm-INVINH4 protein: For transient expression of the Zm-INVINH4 protein in the potato leaves, the coding region was cloned into the pB7WG2 vector in the downstream of the 35S promoter (for primer sequence refer to the Table S1). Gateway cloning technics was used to construct the recombinant Zm-INVINH4 protein expressing vector, and the vector was transferred into *E. coli*. The cDNA of *Zm-INVINH4* was cloned without signal peptide into the pETG10A vector, which leads to the expression of the protein in fusion with a c-terminal 6× His-tag. DNA fragments were purified using the NucleoSpin Extract II Kit (Macherey–Nagel, Duren, Germany), according to the manufacturer’s instructions. A purified product was ligated using T4 DNA ligase (New England Biolabs, Frankfurt, Germany), with incubation at 14 °C for 16 h. A ligation product was transformed into *E. coli* competent DH5α cells by electroporation.

XFP fusions: For expression of the Zm-INVINH4 protein C-terminally tagged with GFP, full-length cDNA of *Zm-INVINH4* was cloned into the pB7FWG2 vector. To obtain a Zm-INVINH1 construct C-terminally tagged with RFP, full-length *Zm-INVINH1* cDNA was cloned into the pB7RWG2 vector.

All primers used in plasmid cloning are presented in Table S1.

### 4.4. Gene Expression Analysis by qPCR

qPCR analysis was performed with the Rotor-Gene Q system (Qiagen) using SYBR Green (S7563, Invitrogen) to monitor dsDNA synthesis. Thermal cycling conditions were identical for all primer pairs: 95 °C/6 min, followed by 40 cycles of 95 °C/20 sec–58 °C/20 sec–72 °C/20 s, followed by a melt cycle from 50 to 95 °C. To determine the primer efficiency, serial dilutions of the templates were conducted for all primer combinations. Each reaction was performed in triplicate, and the amplification products were examined by agarose gel electrophoresis and melting curve analysis. The expression stability of reference genes (*actin* and *ubiquitin*) were assessed using GeNorm algorithms, and the relative gene expression level was calculated by normalizing the geometric mean of the reference genes, according to a previously described method [33,34]. Primers for reference genes and target genes are presented in Table S1. For each tissue, three independent cDNA preparations were analyzed with at least three technical replicates of each.

### 4.5. Expression of Recombinant Zm-INVINH4 Protein in E. coli

Plasmid construct for expression of Zm-INVINH4 protein (see above) was transformed into *E. coli* strain Rosetta–gami (Novagen, Madison, WI, USA). Purification of recombinant protein followed the procedure of Eufinger [35].

### 4.6. Plant Transformation

Transient expression in six to eight week-old potato leaves was performed by *Agrobacterium tumefaciens* strain C58C1 harboring Zm-INVINH4 overexpression plasmids (see Section 4.3) via leaf infiltration, as described in Wolf et al. and Bhaskar et al. [25,26]; transformation with P19 served as a control to account for agrobacterium transformation-induced induction of endogenous invertase activities [25].

### 4.7. Protein Extraction From Plant Material and Determination of Invertase Activity

Extraction of soluble and cell wall-bound proteins from plant tissues was carried out following the protocol described in Link et al. [36]. Bound proteins were eluted from the re-suspended cell wall fraction with 500 mM NaCl for one hour at 4 °C, using an overhead shaker, followed by centrifugation at 10,000× *g* at 4 °C. Soluble and salt-eluted proteins (i.e., from a cell wall-bound fraction) were washed and concentrated by a Centrifugal Filter (50 KDa Amicon Ultra; Millpore, Darmstadt, Germany) with 50 mM NaOAc buffer pH 5. Protein concentrations were determined by a Bradford assay (Roti^®^-Quant; Roth, Karlsruhe, Germany). Different aliquots were incubated with 100 mM sucrose (Applichem, Darmstadt, Germany) in 50 mm NaOAc buffer, pH 5.0 at 37 °C for different time intervals. After incubation, the reaction was stopped by heating at 95 °C for 5 min. Released hexoses were determined by a coupled spectrophotometric enzyme assay as described in Link et al. [36]. All enzyme measurements were performed under conditions where activities were proportional to enzyme amount and incubation time.

### 4.8. Functional Characterization of E. coli-Expressed Invertase Inhibitor Protein

The recombinant inhibitor protein Zm-INVINH4 was purified from *E. coli* extracts (see above) by His-tag affinity chromatography [36]. For inhibition assays, Zm-INVINH4 protein was pre-incubated with inhibitor-containing fractions for 30 min at 30 °C to allow a complex formation with invertase proteins. Thereafter, remaining invertase activities we determined as described above [36]. All enzyme measurements were performed under conditions where activities were proportional to the enzyme amount and incubation time.

### 4.9. CLSM Analysis

Microscopic analyses were carried out using a confocal laser scanning microscope (LSM510 Meta, Zeiss, Jena, Germany). The following excitation and detection wavelength were used: GFP, excitation at 488 nm, detection at bandpass 505–530 nm; RFP, excitation at 543 nm, detection at bandpass 560–615 nm.

### 4.10. Statistical Analysis

Gene expression analysis via qPCR and invertase activity measurement (plant extracts or recombinant proteins) were performed at least 3 independent experiments, with 3 technical replicas for each experiment. For statistical analysis, a Student’s *t*-test using SPSS 20.0 software (SPSS Inc., Chicago, IL, USA) was used. For further details of statistical analysis see figure legends.

## Figures and Tables

**Table 1:** The predicted subcellular localization of Zm-INVINH4.

Program	Apoplast	ER	Golg	CHL	MT	Other
PSORT	0.805 ^a^	0.2	0.100	NA	NA	NA
TargetP	0.642	NA	NA	0.025	0.248	0.006
SignalP	0.931 ^a^	NA	NA	NA	NA	NA

The three intracellular targeting prediction programs used were PSORT (Available online: http://psort.hgc.jp/), Target P (Available online: http://www.cbs.dtu.dk/services/TargetP/), and SIGNAL P (Available online: http://www.cbs.dtu.dk/Services/Signa.lP/). Chloroplast (CHL); endoplasmic reticulum (ER); Golgi body (Golgi); mitochondria (MT); not applicable (NA). ^a^ The higher the value, the higher the probability of localization in the indicated subcellular compartment.

## Supplementary Materials

Supplementary materials can be found at https://www.mdpi.com/1422-0067/20/17/4081/s1. Table S1. Oligonucleotides used for PCR amplification and cloning.
