# Supplementary material for: Functional Characterization of a Drought-Responsive Invertase Inhibitor from Maize (Zea mays L.)
Source: Int J Mol Sci. 2019 Aug 21;20(17):4081. doi: 10.3390/ijms20174081 (PMC6747265; doi:10.3390/ijms20174081)
Supplement: Supplementary file 1 [file ijms-20-04081-s001.pdf]

Supplementary Materials:

Table S1. Oligonucleotides used for PCR amplification and cloning. Included are primers for full length constructs (full); partial cDNAs for qPCR (qPCR); for heterologous expression of mature proteins in *E.coil* (pETG10A); for Gateway 2-step PCR (For pETG10A vector *E.coil* expression).

cDNA	Comment	Primer name	Primer sequence (5'- 3')
Zm-INVINH4	Full gateway +/-Stop codon	Inh4GW-F	GGGGACAAGTTTGTACAAAAAAGCAGGCTCCATG<br>GCAGCAACAAGGGCTTC
		Inh4GW+SC-R	GGGGACCACTTTGTACAAGAAAGCTGGGTCCTAG<br>TAGGTCTCCTGGTTTTGCA
		Inh4GW-SC-R	GGGGACCACTTTGTACAAGAAAGCTGGGTCGTAG<br>GTCTCCTGGTTTTGCA
	qPCR	Inh4qPCR-F	GAGCAACAGAACGCCAAGC
		Inh4qPCR-R	GCGATGCAGAGGTCGTAGAA
	pETG10A	Inh4pETG-F	TATTTTCAGGGCTTCCGCCTCCCCGACTCC
		Inh4pETG-R	AGAAAGCTGGGTGCTAGTAGGTCTCCTGGTTTTG
Zm-INVINH1	Full gateway +/-Stop codon	Inh1GW-F	GGGGACAAGTTTGTACAAAAAAGCAGGCTCCATG<br>AAGCTTCTGCAAGCTCTGT
		Inh1GW-R+	GGGGACCACTTTGTACAAGAAAGCTGGGTCTTAC<br>AACGCGGCCGTTACAGACA
		Inh1GW-R-	GGGGACCACTTTGTACAAGAAAGCTGGGTCCAAC<br>GCGGCCGTTACAGACAGC
	qPCR	Inh1qPCR-F	TGTCAGTCAGGCCAGTCAAG
		Inh1qPCR-R	AGTAGGCGTAGCCGGTGTC
Zm-Ivr2	qPCR	Ivr2qPCR-F	GTTCTACGCGTCCAAGACG
		Ivr2qPCR-R	GTTGCTACCCGTCTTGGTG
Zm-Ivr3	qPCR	Ivr3qPCR-F	TCTCCCTCCTCCCATTG
		Ivr3qPCR-R	CTCCCCTACTCGTACGTG
Zm-INCW1	qPCR	Incw1qPCR-F	GGACCGGTACTACCCCGA
		Incw1qPCR-R	GTCCAGCCAGATCTTCCTTG
Zm-INCW2	qPCR	Incw2qPCR-F	TACCAAGTCGTCCCTGAACC
		Incw2qPCR-R	TAGACCCTGGAGAGGATGC
Zm-INCW5	qPCR	Incw5qPCR-F	GGAGAGGACCGCCGTGT
		Incw5qPCR-R	CGTCCAGAGATGACTTGGTGG
Zm-INCW8	qPCR	Incw8qPCR-F	GGAGGCTGTGGTCAAGGATT
		Incw8qPCR-R	ATCCAACCATATAGTCCTGGGAA
Zm-ubiquitin	qPCR	UbiqPCR-F	CTC TTT CCC CAA CCT CGT GTT
		UbiqPCR-R	ACG AGC GGC GTA CCT TGA
Zm-actin	qPCR	ActinqPCR-F	CGATTGAGCATGGCATTGTCA
		ActinqPCR-R	CCCACTAGCGTACAACGAA
Gateway 2-step PCR pETG10A vector E.Coli expression		attB1_TEV_adaptor	GGGGACAAGTTTGTACAAAAAAGCAGGCTCTGAG<br>AATCTTTATTTTCAGGGC
		attB2 adaptor	GGGGACCACTTTGTACAAGAAAGCTGGGT
